# Myocardial Infarction as an Early Presentation in Thrombotic Thrombocytopenic Purpura: A Rare Case Series

**DOI:** 10.1177/2324709618773789

**Published:** 2018-05-02

**Authors:** Sumit Dahal, Dipesh K. C. Ghimire, Saroj Sapkota, Suyash Dahal, Paritosh Kafle, Manjul Bhandari

**Affiliations:** 1 Interfaith Medical Center, Brooklyn, NY, USA; 2 KIST Medical College and Teaching Hospital, Lalitpur, Nepal

**Keywords:** thrombotic thrombocytopenic purpura, myocardial infarction, cardiac involvement

## Abstract

Renal and neurological involvements are frequently seen in thrombotic thrombocytopenic purpura (TTP). Cardiac involvement, however, has been rarely reported. In this article, we present 2 cases of myocardial infarction in patients with TTP. In the first case, a young man presented with non–ST-segment elevation myocardial infarction that resolved promptly with plasmapheresis. The second patient developed ST-segment elevation myocardial infarction early in the course of the disease and died before plasmapheresis could be initiated. Hence, a high degree of suspicion with prompt diagnosis and treatment is needed to prevent mortality associated with cardiac involvement in TTP.

## Introduction

Thrombotic thrombocytopenic purpura (TTP) is associated with low levels of von Willebrand factor cleaving protease, ADAMTS-13.^1^ ADAMTS-13 deficiency is due to gene mutations in the hereditary form of the disease, and to anti-ADAMTS-13 autoantibodies in the acquired form.^2^ It is characterized by the development of microthrombi in small blood vessels leading to consumptive thrombocytopenia and microangiopathic hemolytic anemia. Renal and neurological involvements are frequently seen. Cardiac involvement, however, is rarely reported, even though the heart continues to be one of the most frequently involved organs at autopsy examination.^3-6^ In this article, we present 2 cases of myocardial infarction (MI) in patients with TTP—first is a case of non–ST-segment elevation MI (NSTEMI) as initial presentation of TTP, while the second is a case of fatal ST-segment elevation MI (STEMI) occurring early in the course of the disease.

## Case 1

A 46-year-old male presented to our emergency department with left-sided chest discomfort associated with blood-smeared sputum and blood in urine for 1 day. There was no similar episode in the past and no family history of any bleeding disorder or malignancy. Vitals were stable at presentation. Physical examination was significant for icterus and absence of any petechial rash, lymphadenopathy, or hepatosplenomegaly.

**Figure 1.**
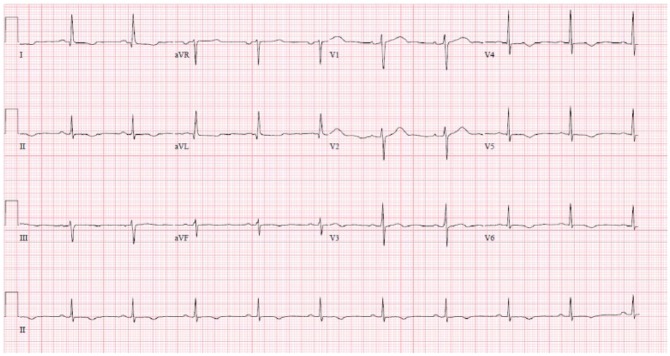
Electrocardiogram of case 1 showing T-wave inversion.

Laboratory tests were significant for leukocyte of 15 000/µL (normal = 4500-11 000/µL), hemoglobin of 10.7 g/dL (normal = 13.5-17.5 g/dL), hematocrit of 32% (normal = 41% to 53%), platelet of 13 000/µL (normal = 130 000-400 000/µL), blood urea nitrogen of 41 mg/dL (normal = 8-20 mg/dL), serum creatinine of 1.9 mg/dL (normal = 0.4-1.3 mg/dL), and total bilirubin of 3.1 mg/dL (normal = 0.3-1.2 mg/dL) with unconjugated bilirubin of 2.6 mg/dL (normal = 0.2-1.1 mg/dL). Serum troponin was elevated at 1.03 ng/dL (normal <0.05 ng/dL), which subsequently trended up to 1.09 ng/dL and 1.75 ng/dL, while the initial electrocardiography (EKG) showed T-wave inversion in lateral leads (V5-V6) with echocardiogram showing lateral wall hypokinesia (Figure 1). A diagnosis of NSTEMI was made, but owing to severe thrombocytopenia, no antiplatelet therapy or other cardiac intervention was done. He was, however, started on amiodarone as he had multiple episodes of nonsustained ventricular tachycardia. Subsequent tests showed an elevated serum lactate dehydrogenase (LDH) level of 1499 IU/L (normal = 98-192 IU/L) and a low haptoglobin level of less than 10 mg/dL (normal = 34-200 mg/dL) with many schistocytes on peripheral smear. ADAMTS-13 activity of less than 10% (normal >66%) with elevated ADAMTS-13 antibody titer of greater than 140 U/mL (normal <12 U/mL) confirmed the diagnosis of severe acquired TTP, and the patient was started on plasmapheresis. Over the subsequent days, his symptoms resolved and there was significant improvement in his platelet count, LDH, serum creatinine, and bilirubin, and his troponin level began trending down.

His hospital course was, however, complicated by a rapid drop in platelet count after skipping a session of plasmapheresis. So a diagnosis of refractory TTP was made, and the patient was put back on daily plasmapheresis schedule and started on weekly rituximab at a dose of 375 mg/m^2^ body surface area. He responded well to rituximab, and the frequency of plasmapheresis was slowly tapered off after 2 doses of rituximab with plans for 2 more doses as an outpatient. He continued to remain asymptomatic while his hematological parameters stabilized with a platelet count of 257 000/µL at discharge.

## Case 2

**Figure 2.**
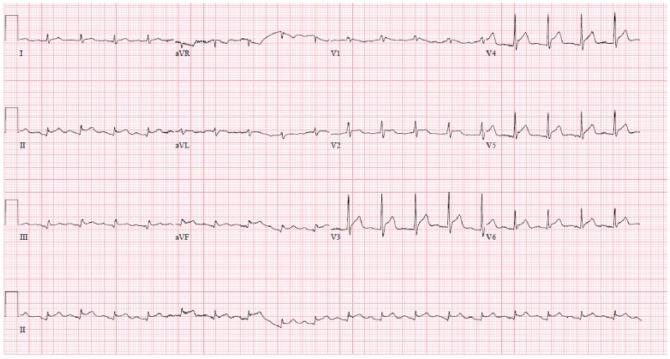
Electrocardiogram of case 2 showing ST-segment elevation.

A 61-year-old male presented with 1 episode of blood mixed stool and 3 days of dark colored urine. He, however, denied any abdominal pain, nausea, vomiting, change in bowel habit, polyuria, pain, or burning on micturition. His past medical history was significant for right knee replacement and lumbar disc herniation. He had no similar episode in the past, and no family history of any bleeding disorder or malignancy. He had no known allergies and took no medications. His vitals were stable at presentation. Physical examination was significant for the presence of mild icterus and absence of any pallor, petechial rash, lymphadenopathy, or hepatosplenomegaly. Laboratory investigations at presentation showed hemoglobin of 13.1 g/dL, hematocrit of 37.3%, platelet count of 8000/µL, serum blood urea nitrogen of 57 mg/dL, serum creatinine of 2.65 mg/dL, and total bilirubin of 4 mg/dL with unconjugated bilirubin of 2.8 mg/dL. The initial EKG showed nonspecific findings. Subsequent tests showed an elevated LDH (2299 IU/L) and a low haptoglobin (less than 10 mg/dL) with many peripheral schistocytes. So with a probable diagnosis of TTP, he was planned for plasmapheresis. Meanwhile, ADAMTS-13 workups were sent, which was later reported as an ADAMTS-13 activity of less than 10% with an elevated ADAMTS-13 antibody titer of 19 U/mL. However, he suddenly developed severe shortness of breath before the planned plasmapheresis. There were widespread ST-segment elevations on his EKG with a serum troponin of 4.12 ng/mL and left ventricular wall hypokinesia on bedside echocardiography (Figure 2). His condition worsened rapidly over the next few minutes, and he developed cardiac arrest and expired.

## Discussion

Our first patient had TTP complicated by NSTEMI at presentation, as evidenced by his left-sided chest discomfort, elevated troponin, EKG changes, and focal ventricular hypokinesia on echocardiogram. Absence of other risk factors for coronary artery disease and the prompt resolution of the episode with plasmapheresis supports TTP as the cause of his NSTEMI. His clinical course was further complicated by episodes of nonsustained ventricular tachycardia. Our second case had TTP complicated by STEMI early in the course of the disease with subsequent cardiac arrest and death.

Cardiac involvement in TTP may be in the form of infarction, congestive heart failure, arrhythmias, or cardiogenic shock.^7,8^ MI in TTP is thought to be likely due to microthrombi from massive platelet aggregation.^9^ Widespread myocardial involvement at autopsy with microthrombosis of the small vessels supplying the myocardium has been reported in up to 77% of the patients dying from TTP.^3-5^ However, MI as a presenting or early feature of TTP is rarely seen, with a reported incidence for any cardiac involvement in TTP being 10% to 40% in a few limited clinical cohorts.^7-14^ This discrepancy between clinical and autopsy findings may account for sudden death resulting from unrecognized cardiac events.

Prompt diagnosis and treatment is necessary as mortality is significantly higher in patients with TTP who have cardiac involvement.^7-9^ A study by Benhamou et al showed that a cardiac troponin I level greater than 0.25 µg/L in patients with TTP was associated with more refractory disease and 3-fold increase in the risk of death.^15^ There is, however, a lack of established guidelines for the management of these cases. The management of TTP-induced MI is challenging as severe thrombocytopenia and accompanying acute renal failure usually precludes any invasive therapy in the form of cardiac catheterization and percutaneous intervention or any medical management in the form of antiplatelet and anticoagulant therapy.^11,16^ Early initiation of plasmapheresis, which involves removing large volume of the patient’s plasma containing any ADAMTS-13 antibody and replacing it with donor plasma with normal ADAMTS-13 activity, is essential to prevent further myocardial damage and associated mortality.^14,17-20^ Furthermore, as evidenced by the above-mentioned cases, continuous cardiac monitoring is vital in patients with TTP given their propensity for developing fatal arrhythmias. Relapsing and refractory cases of TTP like our first case have been effectively treated with rituximab, a monoclonal antibody against CD20 receptors on memory B cells.^20-24^ Further studies are needed to delineate the role, dosing, and frequency of rituximab in TTP, including TTP-related MI.

## Conclusion

MI, thought to be due to microthrombi from platelet aggregation, rarely is a presenting or early feature of TTP. As illustrated by our cases, however, it can be an early complication of TTP. A high degree of suspicion and prompt diagnosis is necessary, because mortality is significantly higher in patients with TTP who have positive cardiac biomarkers. Early initiation of plasmapheresis is essential to reduce mortality.
